# An international initiative to improve mental healthcare in Palestine through a tripartite collaboration

**DOI:** 10.1192/bji.2025.8

**Published:** 2025-08

**Authors:** Leen Farouki, Nadia Dabbagh, Richard Braithwaite, Samah Jabr, Agnes Raboczki, Vivek Agarwal, Susannah Grant, Mohammed Al-Uzri

**Affiliations:** 1 Royal College of Psychiatrists, London, UK; 2 Division for Paediatric Mental Health, Dubai Health – Al Jalila Children’s Hospital, Dubai, UAE; 3 Committee for Electroconvulsive Therapy & Related Treatments, Royal College of Psychiatrists, London, UK; 4 Sussex Partnership National Health Service Foundation Trust, Worthing, UK; 5 Mental Health Unit, Palestinian Ministry of Health, Ramalla, State of Palestine; 6 George Washington University, Washington, DC, USA; 7 Norfolk and Suffolk NHS Foundation Trust, Norwich, UK; 8 Health Sciences Department, University of Leicester, Leicester, UK

**Keywords:** Mental health, international collaboration, tripartite model

## Abstract

The number of people living in Palestine with mental disorders is significantly higher than the global average. Military occupation, violence and poverty contribute to collective trauma. International agencies have emphasised the need for collective action and systemic solutions. The Ministry of Health in Palestine, Medical Aid for Palestinians and the Royal College of Psychiatrists have collaborated to develop a national strategy for child and adolescent mental health, enhance psychiatric training and improve electroconvulsive therapy provision. The article details how this collaboration has demonstrated partnership and local ownership to empower Palestinian communities to make sustainable mental healthcare improvements.

There is a significant gap in mental health service provision in Palestine. In comparison with other Eastern Mediterranean countries, where the prevalence of mental disorders already exceeds global averages, Palestine bears the highest burden, with over 1 million people requiring services.^1,2^ The nearly 60-year military occupation, along with violence, poverty and other social and political issues that collectively affect communities, emphasise the need for systemic approaches to mental healthcare delivery in Palestine.^3^

The World Health Organization (WHO)’s QualityRights initiative aims to promote the rights of people with mental health conditions while improving quality of care.^4^ An aligned WHO initiative, the Mental Health Gap Action Programme, emphasises human rights and details the approach to scaling up, or building capacity, for mental healthcare.^5^ Scaling up is defined as the joint responsibility of governments, healthcare professionals, civil society, families and local and international communities.^6^ This approach involves building partnerships for collective action,^5^ echoing the Alma-Ata Declaration, which encourages countries to ‘cooperate in a spirit of partnership’ to ensure healthcare for all.^7^

This ethos of cooperation while scaling up, promoting rights, and improving quality of care is demonstrated by the collaboration between the Ministry of Health in Palestine (MoH), the charity Medical Aid for Palestinians (MAP) and the Royal College of Psychiatrists (RCPsych) in the UK, stemming from a participatory needs assessment. These three organisations worked together in Palestine in 2022 to produce a national strategy for child and adolescent mental health, improve the education and assessment of trainee psychiatrists, develop the provision of electroconvulsive therapy (ECT) and provide training on safe breakaway techniques. The collaboration and chosen priority areas were requested and determined by the Palestinian partners at both MoH and MAP. RCPSych was able to offer expertise in these diverse priority areas through upholding the principles of global mental health, as enshrined in its international strategy.^9^

## Stakeholders

### MoH

MoH provides healthcare in the West Bank. Within MoH, the Mental Health Unit is currently headed by Dr Samah Jabr (S.J.), a champion of decolonial, culturally informed approaches to mental health.^8^

### MAP

MAP is a charity that works together with communities to improve access to healthcare and develop healthcare skills and knowledge among local providers of mental health and psychosocial support. MAP endorses a holistic approach, addressing both symptoms and their source by supporting communities and individuals in coping and resilience while also addressing contextual factors. This approach reflects the values of empowering local communities to heal and rebuild by acknowledging social issues, including healthcare access, poverty, trauma, settler violence and threats of home demolition, as the root of many mental health problems.^9^

### RCPsych

RCPsych’s international strategy enables members to support and deliver mental healthcare in different global settings. It commits to partnership in mental healthcare by requiring interest and acceptance from local ministries and partners, local sustainability in projects, respect for local culture and peoples, non-complicity in systemic stigma that disadvantages certain groups and upholding human rights.^10^

## Principles of the work

RCPsych’s international strategy^10^ requires that collaborations adhere to three principles: tripartite partnership, ownership and sustainability.

### Tripartite partnership

Collaboration between a host on the ground, a funding or technical organisation providing logistical support and RCPsych, bringing expertise in training and education.

### Ownership

Ensuring local control over project direction, based on the local understanding of healthcare needs and cultural context.

### Sustainability

Focusing on long-term, maintainable improvements beyond the project’s initial scope, including sustainable funding.

## Description of the work

The collaboration started with provision of supervision sessions by volunteers from RCPsych to psychiatric trainees in Palestine as part of their training to become qualified psychiatrists. However, the collaboration expanded to include (a) a national strategy for child and adolescent mental health in Palestine, (b) examinations and (c) provision of ECT.

### National strategy for child and adolescent mental health in Palestine

The three RCPsych international strategy principles were interwoven into the development of this strategy, which aimed to provide direction, align activities and utilise limited resources and funds effectively. RCPsych made cultural considerations by inviting a child psychiatrist (N.D.T.) of Palestinian background with extensive experience in trauma and suicide research. She approached the team of MoH and MAP advisors, led by the Head of the MoH Mental Health Unit (S.J.), who outlined their requirements for the strategy. A series of stakeholder thematic meetings took place to allow local organisations decide who would be represented in the development of this project, and the strategy’s direction and themes. The psychiatrist took an anthropological approach to understanding the issues and contexts described by the advisors. This, in conjunction with taking a participatory approach prioritising feedback from local professionals, ensured a co-produced document guided by local professionals and facilitated by the external expert, N.D. The strategy sets out the key priorities for child and adolescent mental health so that funders, institutions, organisations and community members can align their activities in a coordinated and efficient manner.^11^ The strategy addresses the fundamental underlying political, economic and social drivers that affect Palestinian children’s mental health and well-being.

### Examinations

The chair of the RCPsych panel on Clinical Assessment of Skills and Competencies (V.A.) and RCPsych’s Head of Examinations (S.G.) worked with both MAP and MoH with regard to the examination and assessment of psychiatrists in Palestine. Ahead of their visit, local teams identified areas for development, namely oral examinations, the syllabus and question writing. MAP organised the mission, including pre-visit preparation and a comprehensive programme. This reflected the value of ownership, because local teams were in control of planning and coordinating activities. RCPsych team members led a workshop on examination formats and processes, which was attended by the Director General of the Palestinian Medical Council, psychiatry representatives and medical professionals from other departments involved in training and examinations. Delegates were highly engaged and contributed by discussing how the RCPsych international strategy principles presented could be adapted and applied locally, demonstrating the values of co-production and sustainability.

### ECT provision

RCPsych supports psychiatric training in Palestine by providing education sessions on topics delivered by expert volunteers. During a supervision session, local doctors explained that they felt the process and management of ECT were not serving the best interests of their patients. Without an ECT protocol to refer to, they felt disempowered to apply the required standards of care for the delivery of this treatment. Discussions between the three partners led to an agreement to work together towards this goal. MoH committed to developing a new protocol for ECT provision that is consistent with international standards, and to releasing staff for training. MAP purchased a new ECT machine for Bethlehem Psychiatric Hospital and provided logistical support to the chair of the RCPsych’s ECT Committee (R.B.), who provided the training and gave expert advice on the development of the protocol and specifications for equipment. This project will be described in more detail in a separate publication.

## Conclusion

In Palestine, mental health and the provision of mental healthcare services face difficulties that are exacerbated by the social and political challenges of a century marked by political violence and occupation. Ameliorating these circumstances requires a collective and systemic approach as demonstrated by the present collaboration, which emphasises partnership, local ownership and sustainability in developing and implementing mental health strategies. This can provide immediate support, long-term improvements and local autonomy in service delivery.

Key lessons from this collaboration include the importance of community-centred and culturally informed approaches. Moreover, the importance of participatory approaches and the inclusion of collaborators with diverse portfolios allowing for targeted, multifaceted interventions should be emphasised. Future research could explore the scalability of tripartite partnership methods in other conflict-affected regions. and the long-term impact of participatory approaches on mental health outcomes.
